# Albumen Quality of Fresh and Stored Table Eggs: Hen Genotype as a Further Chance for Consumer Choice

**DOI:** 10.3390/ani11010135

**Published:** 2021-01-10

**Authors:** Chiara Rizzi

**Affiliations:** Department of Agronomy, Food, Natural Resources, Animals and Environment, University of Padova, 35020 Legnaro, Padova, Italy; chiara.rizzi@unipd.it

**Keywords:** hens, dual-purpose, egg, albumen quality, storage

## Abstract

**Simple Summary:**

Consumer interest in food products and their origins is increasing. Knowledge of egg production and quality of purebred hens during the productive period is required for a niche market sustaining and encouraging biodiversity and the peculiarities of the products that consumers can appreciate. Egg production and quality of the eggshell and albumen in fresh and stored eggs of two Italian dual-purpose purebreds (Ermellinata di Rovigo (ER); Robusta maculata (RM)) and two hybrid genotypes (Hy-Line Brown (HB); Hy-Line White (HW)) reared outdoors were compared throughout the laying period. RM breed (brown eggshell) showed fresh and stored eggs with a good eggshell thickness, and Haugh Units (HU) quite stable along the production period; RM total egg mass was lower than ER (light brown eggshell) which showed fresh and stored eggs with more variable HU, due also to a possible effect of lower eggshell thickness and pigmentation, and shape index. The hybrids produced a higher total egg mass than the purebreds and showed an intermediate variation of the egg quality, with HU higher than those of ER and RM only in 1 d eggs, but not in stored eggs.

**Abstract:**

The quality of fresh (1 d) and stored (7–14–21 d, 21 °C) eggs was studied in Italian dual-purpose breeds (Ermellinata di Rovigo (ER), Robusta maculata (RM)) and hybrids (Hy-Line Brown (HB), Hy-Line White36 (HW)), reared outdoors (4 m^2^/bird) and fed commercial feed. The eggs were analyzed at 4 ages, throughout different seasonal environmental conditions, from summer (31, 35 weeks; 25 °C) until autumn (39, 43 weeks, 15 °C). Each genotype showed significant (*p* < 0.01) changes in egg quality. In 1 d eggs, the eggshell thickness changed in RM and HW (quadratic), decreased linearly in ER; Haugh Units (HU) changed (ER–cubic) and decreased (hybrids-linear). In 7 d and 14 d eggs, HU linearly (*p* < 0.01) decreased, except in RM. In 21 d eggs, HU (ER linear decrease; HB, HW quadratic) changed. Significant negative correlations between albumen pH and height were seen in ER (at 1 d, 14 d, 21 d) and HW (at each storage time) eggs, and in RM and HB only in 1 d eggs. RM showed a quite stable albumen quality and a lower total egg mass than ER which showed a more variable albumen quality, due also to a lower eggshell thickness and shape index. The hybrids produced a higher total egg mass than the purebreds and showed an intermediate variation of the egg quality, with an albumen quality higher than those of ER and RM only in 1 d egg, as a result of a higher albumen weight.

## 1. Introduction

The production and quality of table eggs changes according to commercial strain and age of the hens and to rearing conditions [1]. Broadly, consumers choose eggs considering one or more factors such as size, eggshell colour, yolk colour, freshness, but also rearing system and price [2,3]. The albumen egg proportion and its quality usually are not indicated on the table egg cartons, but they are important as these parameters affect the quality of some cooking preparations. The physical aspect of albumen such as its firmness can be evaluated directly and visually by the consumer, whereas Haugh Units (HU) are a more technical parameter which considers the height of albumen and the weight of the egg [4]. All these parameters are useful to give indications to the consumer on the quality of albumen of fresh and stored eggs. The changes of albumen quality during storage depend on the environmental conditions, such as temperature and relative humidity, as they affect the internal reactions of an egg and its final quality: in fact, there are significant differences upon the degradation processes of the egg components between storage at 16 and 30 °C [5]. Relevant factors affecting the albumen secretion are those such as the interactions between environment and the hen physiology and age, involving also the gene expression and protein secretion [6,7,8,9]. Also the egg characteristics such as shape index, eggshell colour and thickness may be involved in the albumen quality during storage [4,10]. Furthermore, in the countryside tourism is increasing year by year and tourists are interested in knowing the typical and historical animal breeds and food production of a region, and thus the origin of a food product has become an important factor as it affects its quality. In Italy, some regions have a historical tradition for poultry breeding based on many chicken breeds differing for phenotypes and productions [11,12,13,14]. For the purpose of farm animal welfare [15,16,17] and biodiversity, the purebred and dual-purpose hens and their eggs may represent a niche production for the farmer and a further opportunity of choice of table eggs for the consumer.

Knowledge of the daily egg mass and egg quality of purebred hens during the productive period is required for sustaining a niche market and encouraging biodiversity and the peculiarities of the products that consumers can appreciate. A previous work compared the egg chemical composition and sensory profile of eggs laid by hybrid and purebred hens; it reported that the albumen height and HU of fresh and stored eggs were similar among hybrids and Italian breed Robusta maculata, whereas the stored eggs of Italian breed Ermellinata di Rovigo showed lower HU [18]. A recent work on the aforementioned genotypes studied hen’s behaviour at laying and the effect of age on the quality of fresh eggs throughout the laying cycle [19].

The aim of this trial was to study the changes according to the age of the hens of some quality parameters of fresh and stored eggs laid by these Italian purebreds and commercial hybrid genotypes reared under outdoor conditions throughout the first phase of oviposition.

## 2. Materials and Methods

### 2.1. Ethical Statement

The research used eggs produced by hens coming from a rearing farm of the Veneto region, according to the principles stated in EC Directive 86/609/EEC.

### 2.2. Genotypes and Rearing Conditions of the Hens

The eggs used for the trial became from two Italian local chicken breeds and two hybrid strains. The Italian breeds were Ermellinata di Rovigo (white plumage and black with white edge hackle and saddle; black main tail feathers and white primaries with black extremities (ER)) and Robusta maculata (silver plumage with black tail and white breast feathers, and white primaries with black extremities (RM)); they are defined as dual-purpose and slow growing breeds and produce brown eggshell eggs. Their origin is from Veneto region (Northern Italy), where they were created during the 1950s, using Sussex and Rhode Island (ER breed) and Brown Orpington and White America (RM breed) [11]. The commercial hybrid strains were Hy-Line Brown (brown plumage and brown eggshell (HB)) and Hy-Line White 36 (white plumage and white eggshell (HW)).

The hens were reared on the same trial station of the Veneto region under outdoor conditions. All the chicks were kept indoors during the first 4 weeks of life, on litter, under infrared radiation lamps, at an environmental temperature daily decreasing from 32 until to 24 °C. At 2 months of age the birds had free access to outdoor spaces. Each genotype had access to indoor (0.20 m^2^/bird) and outdoor (4 m^2^/bird) spaces, divided by netting. In the indoor spaces, the floor was covered by a mixture of straw and wood shavings; on the floor, nests and perches were available to the birds. The animals were given ad libitum two feeds: a first commercial one, throughout the body growing period (0–16 weeks of age) (average composition, % as-fed basis: crude protein = 19.4, metabolizable energy = 11.8 MJ/kg), and a second one, throughout the pubertal age and laying period (17–44 weeks of age) (average composition, % as-fed basis: crude protein = 19.0, fats = 5.0, Ca = 3.9 and *p* = 0.7, lysine = 0.9, methionine = 0.3, metabolizable energy = 11.9 MJ/kg). The birds were subjected to the same prophylaxis procedures, rearing conditions (temperature, photoperiod) and feeding from the time of hatching until the end of the experimental period. The experimental period started when all the four groups were laying (25 weeks old) and lasted until 44 weeks of age.

Throughout the trial, the environmental temperature and the relative humidity levels were checked by a thermo-hygrograph (model TIG-ITH, LSI, Milano, I) (Table 1). Throughout the laying period, the photoperiod was 16L:8D, initially natural according to the seasons and the geographical position of the trial station (Northern Italy), and then it was complimented by artificial light inside the rooms where the birds spent the night.

The hens had free access to the outdoor space where they used to stay throughout the day; the indoor space was used for laying eggs, on rainy days and during the night.

### 2.3. Data Collection

The hens were weighed at the beginning and at the end of the trial. Throughout the laying period, from each genetic flock (ER, HB, HW = 70 hens; RM = 60 hens) the hen-day egg production (number of eggs/number of live hens × 100) and the mean daily weight of the egg (average based on the total daily eggs considering samples of 30 eggs maximum per each genotype) were calculated per each group. The hen-day egg mass was calculated as hen-day egg production (%) × daily egg weight (g). The cumulative egg mass was calculated as the sum of hen-day egg mass values throughout each laying period (4 weeks/period). The egg mass/body gain was calculated as the ratio between the total cumulative hen-day egg mass and the body gain, from 25 until 44 weeks of age. At 43 weeks of age, on samples of 30 eggs per each genotype, the eggshell colour of 1 d egg and the egg dimensions were checked. The eggshell colour was tested by a colorimeter (Chroma meter CR 300 (Minolta Co Ltd., Osaka, Japan), using the CIE [20] scale: the L value reflects lightness (0 = black, 100 = white). The shape index was calculated as the ratio between the width and the length of the egg measured with callipers (0.01 mm). At regular 4-week intervals (31, 35, 39 and 43 weeks of age) samples of 20–30 eggs (depending on the daily production of each genotype) from a whole day’s production were collected, excluding the defective eggs (double yolk, abnormal shell), per each genotype and each storage time (1 day = 1 d, 7 days = 7 d, 14 days = 14 d and 21 days = 21 d); for the egg storage, a room with a constant temperature of 21 °C and 64% relative humidity was used. The 1, 7, 14, and 21 d eggs were weighed and then broken and albumen and yolk were put on a square glass plate, for measuring the albumen height by means of a micrometer (0.01 mm) (Mitutoyo Co, Kawasaki, Japan). The albumen pH was measured by means of pH-meter. The eggshell thickness was measured with a digital calliper (0.001 mm) (Mitutoyo, Japan). For 1 d eggs, the yolk was manually separated from the albumen, weighed and the albumen weight was calculated as the difference between the weight of the egg and the sum of the weight of yolk and eggshell (after drying at 45 °C per 12 h). Egg weight and albumen height were used for calculating the Haugh Units (HU) [4].

### 2.4. Statistical Analysis

The hen-day egg mass (at each age), the eggshell colour (lightness) and the shape index (both at 43 weeks of age, on 1 d eggs), the percentage changes of albumen height and HU throughout 2 storage time intervals (from 1 to 7 days, and from 7 to 21 days; means referred to each age), the HU of 1 d and 21 d eggs were subjected to analysis of variance (ANOVA) considering genotype as main effect using the proc GLM of SAS (SAS Institute Inc., Cary, NC, USA).

The data on 1 d (egg weight, eggshell thickness, eggshell weight, albumen weight, albumen height, HU), 7 d (albumen height, HU), 14 d (albumen height, HU) and 21 d (egg weight, albumen height, HU) eggs of each genotype were evaluated by ANOVA considering age as main effect using the proc GLM of SAS. Furthermore, for each genotype, on the data of 1 d and 21 d egg weight per each age an ANOVA was carried out considering the storage time as main effect using the proc GLM of SAS.

Significant differences among least squared means were tested using Tukey’s test.

For testing linear, quadratic and cubic trends, contrast statements were undertaken using orthogonal polynomial coefficients.

Pearson’s correlations between albumen pH and albumen height in 1 d, 7 d, 14 d, and 21 d eggs of all ages were calculated for each genotype (SAS Institute Inc., Cary, NC, USA).

## 3. Results and Discussion

### 3.1. Environmental Conditions and Egg Yield

Before discussing on the effect of genotype and age on the egg yield and quality, it is worth remembering the rearing environmental conditions, given that they are particularly relevant for the physiology of the hens living under outdoor systems [16]. Table 1 resumes the environmental conditions where the trial was carried out, the cumulative egg mass, the daily laying rate (indicated as hen-day egg production), at the beginning of the trial and throughout the total period, and the egg mass to body gain ratio of each genetic group. In this trial, the period until 30 weeks corresponded to the hottest weeks, when the highest environmental temperature was recorded. Then, the temperature decreased to more suitable values until the last weeks, when it averaged 10 °C, a value under the range of thermal neutrality for chickens, but not considered as a stressing condition for hens living outdoors (Table 1). Anyway, the effect of the environmental temperature on the feed consumption for meeting the changed energy requirements according to the season is known for layer genotypes [21], but the adjustments in the level of critical nutrients are not well known [22]. Furthermore, the pasture was good during the growing period of the birds, but it was poor throughout the laying period, because of the scarcity of rain during summer and the walking activity of the hens which compromised the grass re-growth.

**Figure 1:**
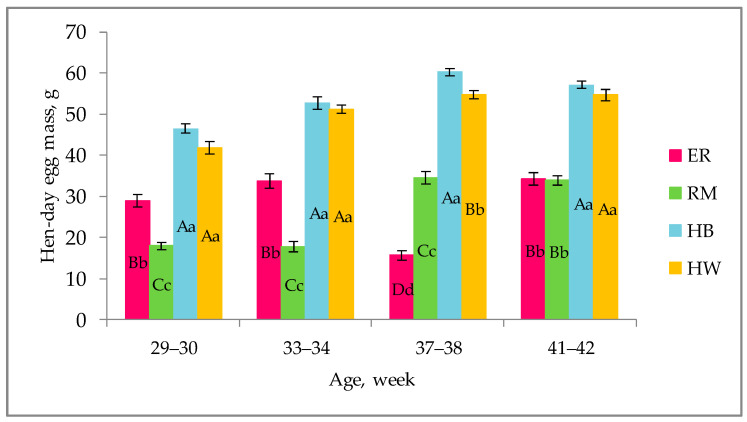
Effect of genotype ^1^ on the hen-day egg mass (lsmeans ± standard error (SE)) throughout the laying period (weeks of age). Different letters among columns for each age indicate different values. a, b, c, d: *p* < 0.05; A, B, C, D: *p* < 0.01. ^1^ Genotype: ER: Ermellinata di Rovigo; RM: Robusta maculata; HB: Hy-line brown; HW: Hy-line white. Observations (*n*) per each age (29–30 weeks, 33–34 weeks, 37–38 weeks, 41–42 weeks): ER (14), RM (14), HB (14), HW (14).

The effect of genotype on the hen-day egg mass, referred to the last 2 weeks until the age for testing the egg quality, is shown in Figure 1.

For each age, the purebreds showed hen-day egg mass values significantly (*p* < 0.01) lower than those of the hybrids; ER was higher (*p* < 0.01) than RM until 34 weeks, it was lower (*p* < 0.01) at 37–38 weeks and then they were similar. The hybrid strains showed similar values, except at 37–38 weeks, when HW was lower (*p* < 0.01) than HB.

**Figure 2:**
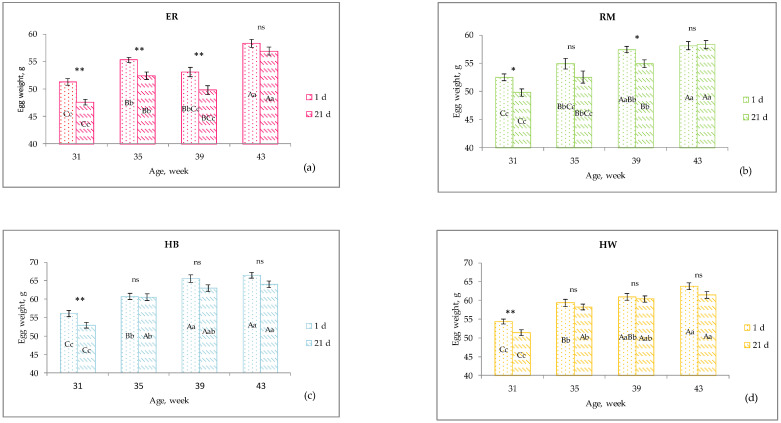
Effect of age on the weight of 1 d egg (lsmeans ± SE) and stored 21 d eggs (lsmeans ± SE) of each genotype ^1^. Different letters among similar columns (storage time) indicate different values. a, b, c: *p* < 0.05; A, B, C: *p* < 0.01. *: *p* < 0.05 and **: *p* < 0.01 indicate different values between columns of different storage time per each age. ns: no significance. ^1^ Genotype: ER: Ermellinata di Rovigo (**a**); RM: Robusta maculata (**b**); HB: Hy-line brown (**c**); HW: Hy-line white (**d**). Observations (*n*) per age (at 1 d; at 21 d): ER (30, 30, 24, 30; 30, 24, 18, 20), RM (25, 22, 22, 24; 20, 16, 20, 20), HB (30, 30, 24, 30; 29, 25, 25, 25), HW (30, 30, 24, 30; 28, 24, 24, 24).

The hen-day egg mass differed among the genetic groups, as a consequence of the different oviposition rate and egg weight (Figure 2) throughout the monitored laying period, and a tendential increasing trend of laying activity was seen. The purebreds exhibited a more variable trend with decreasing values corresponding to a decreased laying activity: the two purebreds showed a drop at 33–34 weeks (RM) and at 37–38 weeks (ER). As far as RM is concerned, given that after 30 weeks the hens showed a drop in laying rate and a consequent decrease of the hen-day egg mass, in Figure 1 the increasing trend is appreciable between 29–30 and 37–38 weeks. The ER and RM, as dual-purpose breeds, are characterized by slower body growth in comparison to that of hybrid hens as well as by a higher body size [23]: these physiological conditions do not allow the purebred birds to show egg production curves similar to those of hybrids in terms of egg quantity and absence of fluctuations. In laying hens these two items reflect the overall body conditions and physiological requirements which, in dual-purpose breeds and especially throughout the first months of laying, involve also the muscle growth [23]. As reported, the Hy Line hens complete the muscle growth earlier than the purebreds, at about 30 weeks of age [24]. Furthermore, a more variable laying rate, throughout the productive period, as generally shown by purebreds, allows the hens to have a healthy condition of their bones: such a skeletal mineral status is an important issue for the welfare of laying hens [25]. As far as the 41- to 42-week period is concerned, Figure 1 shows a light decrease of values of RM, HB and HW; such a decrease may be due to the low environmental temperature and higher body thermal regulation requirements, but also an age effect could be considered. The oviposition curve is well known for hybrids reared under controlled environmental conditions [24], whereas for the purebreds there is a lack of data; for these purebreds, literature reports a total egg production approximately less than half of the total number of eggs produced by the hybrids [11]; furthermore, hybrids have been selected for an early onset of laying, at about 20 weeks of age, and an early peak of production [24].

### 3.2. Egg Quality

In Figure 2, the effect of age on fresh and stored egg weight per each genotype is shown. For 1 d egg, the effect of age was significant (*p* < 0.01) for all the genotypes (Figure 2a–d), which showed increasing egg weights throughout the studied period. The weight of the ER eggs increased more slowly than those of the other groups; ER hens are dual-purpose birds, as well as the RM hens, but ER have a lower body weight (1.3 vs. 2.0 Kg) and higher hen-day egg production, as a consequence of earlier onset of laying (Table 1), and an interaction between body physiology and environmental conditions may occur. An important trait for the egg quality is the weight: the four genotypes produced eggs classifiable into different size classes, according to the EC regulation [26]: the ER and RM eggs were small size (<53 g) until 39 and 35 weeks of age, respectively, and then medium size (53–63 g); the hybrid eggs were medium size at all the ages. For the HB and HW hens, egg weights of large size (>73 g) were observed at 39 and 43 weeks of age, and at 9.3% and 5.2% of the daily production, respectively [19].

**Figure 3:**
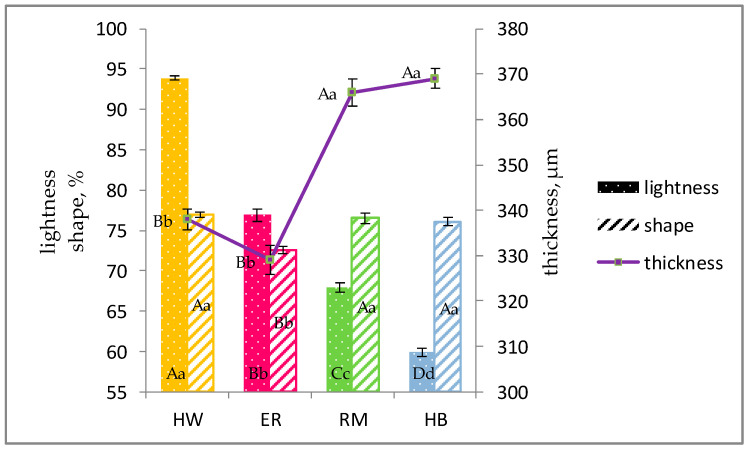
Effect of genotype ^1^ on eggshell lightness ^2^, shape ^3^ and thickness ^4^ (lsmeans ± SE). Different letters among similar columns (lightness = points, shape = diagonals) and on the line (thickness) for each genotype indicate different values. a, b, c, d: *p* < 0.05; A, B, C, D: *p* < 0.01. ^1^ Genotype: ER: Ermellinata di Rovigo; RM: Robusta maculata; HB: Hy-Line Brown; HW: Hy-Line White 36. ^2^ Observations (*n*): ER (30), RM (30); HLB (30); HLW (30). ^3^ Observations (*n*): ER (35), RM (34); HLB (36); HLW (34). ^4^ Observations (*n*): ER (111), RM (96); HLB (119); HLW (118).

In Figure 3, the effects of genotype on the eggshell lightness, shape index and thickness are shown. The eggshell lightness significantly (*p* < 0.01) decreased according to the HW, ER, RM and HB genotype; the ER eggs showed the lowest (*p* < 0.01) shape index when compared to the other groups. The eggshell thickness was higher (*p* < 0.01) in RM and HB than in ER and HW. The hens laid eggs with different eggshell colour: the HW eggs have white shells, whereas the eggs of the other three genotypes have brown shells, but with a different colour intensity. The redness and yellowness index significantly (*p* < 0.01) differed among the brown-egg groups and gradually increased in ER (a* = 9, b* = 22, respectively), RM (a* = 12, b* = 25, respectively) and HB (a* = 17, b* = 29, respectively) [19].

Before discussing the results on some egg quality traits according to the age of the hens, it is worth stressing that the birds belonged to genotypes selected for meat and egg production (dual-purpose breeds) and egg production (hybrids), showing different body growth and oviposition rate, as indicated by the egg mass to body gain ratio in Table 1. Each genotype showed different cumulative egg mass produced until each of the four studied ages (Table 1), thus this last aspect has to be considered also. The double-purpose and slow-growing genotypes show, in comparison to the hybrids, selected for high egg production and with earlier skeletal and muscle growth [24], a concomitant body weight gain and egg production, thus the nutrients may be addressed to a different extent to body tissues and to the ovary and oviduct for the egg formation. The four groups showed different egg mass to body gain ratios, and ER and HW hens showed a ratio double that of RM and HB, respectively.

The effect of age on the eggshell thickness and weight, and albumen weight throughout the laying period is shown in Table 2. The eggshell thickness decreased in ER (*p* < 0.05) at 39 weeks and in RM (*p* < 0.01) at 35 weeks, and thereafter increased. It did not change in HB, whereas HW showed a decrease at 35 weeks and then a gradual increase until 43 weeks (*p* < 0.05). For ER a significant (*p* < 0.01) linear decrease was observed, whereas RM (*p* < 0.01) and HW (*p* < 0.01) showed a quadratic trend. The eggshell weight significantly (*p* < 0.05) increased in all the genotypes, showing a linear (*p* < 0.01) trend.

These results are a consequence of the known increase of the egg size and its components according to the age of the hen [19], but they may be due also to the decrease of the environmental temperature conditions checked throughout the laying period studied. In fact, in heat-stressed hens an alkalosis condition may occur and induce a decrease of mineral deposition on the eggshell [27]. The eggshell formation starts in the isthmus, where the eggshell membranes, arranged in fibrous outer and inner layers, constitute the nucleation sites for the initiation of eggshell mineralization. Collagens are the fibrous components of the eggshell membrane and the expression of collagenX (COL10A1) mRNA is higher in this tract of oviduct of laying hens [28]. Many matrix proteins are responsible of the organization of the calcite crystals during eggshell calcification and other proteins of the uterus epithelium have a role in ion-regulation across the epithelium for the mineralization of the egg. The huge amount of Ca^2+^ ions required for eggshell mineralization is continuously supplied to the uterus from the bloodstream where ions come after mobilization from the medullary bones under the influence of estrogen [29]. Differences in hormonal profile and expression of several genes, most of them recently found, may be factors responsible for differences of eggshell quality among genotypes, as they are involved in the eggshell membrane formation and in the Ca^2+^ transportation in the uterus for the eggshell biomineralization [28,29]. A further important concern for the shell formation is the calcium provision rate, directly by the diet or indirectly by bone resorption, which may differ among the genotypes according to the time of the last feed intake, and to some physiological conditions of the hens [30]. During the eggshell calcification, laying hens cover, partially, the demand of calcium with increased mobilization from the medullary bone, as a labile calcium source [31,32], which is accompanied by a decrease of cancellous bone volume, under the influence of estrogen. The majority of studies have investigated eggshell formation and bone stability in hybrid hens, and the number of studies carried out on chicken purebreds is very limited. Recently, other authors [33] stated that relevant phenotypic differences exist among local chicken breeds, commercial layers and their crosses in terms of bone traits; furthermore, for the genetic groups a rather weak relationship between laying performance, especially in terms of total eggshell production, and bone stability, were found [34]. The aim to improve the impaired bird welfare due to skeletal disorders in highly productive hens [25] needs more knowledge on bone traits of purebred genotypes for their further use in crosses also with hybrid lines.

The albumen weight (Table 2) increased along the laying period: ER increased (*p* < 0.01) at 43 weeks, and RM after (*p* < 0.01) 35 weeks, HB increased (*p* < 0.01) at 35 weeks, HW showed a more gradual significant (*p* < 0.05) increase. A significant (*p* < 0.01) linear component both for the purebred hens and the hybrids was seen. These genotypes showed different proportions of each egg component, as hybrids showed 25% yolk, 64% albumen, whereas ER and RM showed higher yolk (29%) and lower albumen (60%) than those of hybrids; the eggshell proportion of the commercial strains (10.6%) was higher than ER (10%) and lower than RM (11%) [18]. From 31 until 43 weeks, under variable environmental conditions, the yolk percentage linearly increased and the albumen percentage linearly decreased in all genotypes, whereas the eggshell percentage showed a cubic trend in ER, RM and HW and a linear decrease in HB [19]. The albumen composition is critical for the survival of the chicken embryo, by a wide defense activity, but it is important also for the food industry and the consumer which exploit and use many proteins for their technical and nutritional properties [35,36]. The albumen contains nearly 148 proteins: the main is ovoalbumin, a structural protein (about 54% of the total albumen protein), followed by conalbumin, ovomucoid, ovomucin, lysozyme, and others. In the magnum, relaxin (RLN3) provokes the secretion of proteins from the epithelial cells and the expression of RNL3 mRNA is increased by the presence of a yolk. Differences in gene expression caused by intrinsic and extrinsic factors may be responsible for the differences in albumen secretion existing among these genotypes [28].

### 3.3. Albumen Quality of Fresh and Stored Eggs

As the four genotypes showed eggs with different albumen weight and percentage on the egg weight, changes of both albumen and HU according to the age were considered, as the trends may differ.

Table 3 shows the effect of age on the albumen height and Haugh Units of 1 d eggs of each genotype. The albumen height showed variable trends with significant changes in ER (*p* < 0.01), HB (*p* < 0.01), and HW (*p* < 0.05) according to age. RM did not change throughout the experimental period. For ER the albumen height was higher in the presence of low hen-day egg mass, at 31 weeks and 39 weeks; the effect of the summer temperatures, the oviposition rate and factors involved with body growth could have negatively affected the albumen height at 35 and 43 weeks of age. HB decreased after 39 weeks, and HW after 35 weeks, after the summer period, two ages corresponding to an increase of oviposition rate. ER changed following a cubic trend (*p* < 0. 01), for HB a quadratic trend (*p* < 0.01) and for HW a linear trend (*p* < 0.05) were seen.

As well as the HU (Table 3), ER showed an increase at 39 weeks (*p* < 0.01) and a drop at 43 weeks (*p* < 0.01) according to a cubic trend (*p* < 0.01), and RM unchanged throughout the period. HB showed lower values (*p* < 0.01) at 43 weeks and HW showed a decrease from 39 weeks, both of them according to a linear trend (*p* < 0.01).

It is worth remembering that when the hens of different genotypes are reared outdoors, the effect of age on the physiological responses has to be considered together with the rate of oviposition and with diet and other environmental conditions, as already stated. In the experimental conditions, the age of 31 weeks corresponds to a period with the highest environmental temperature. Furthermore, at each age the hens of each genetic group showed a different total egg mass, as a response to different ovary activity and ovulation rate, as well as different activity of the oviduct for the production of albumen, eggshell membranes and eggshell. The decrease of the eggshell thickness could be attributed also to the initial high environmental temperatures and their influence on feed intake, hormonal profile, mineral body condition and other factors (divergent requirements for the body tissues growth and egg production) which have induced also a decrease of oviposition rate and hen-day egg mass (ER at 37–38 weeks, RM at 33–34 weeks). It is well known that elevated temperatures reduce performance in laying hens, particularly when accompanied by high relative humidity [27]; the birds pant, and heat transmission is achieved by evaporative cooling mechanisms from the respiratory tract, and sometimes an excessive blood gas exchange can induce alkalosis that affects the eggshell quality. As concerns the genotype effect, in a previous work the ER hens have reached an egg mass higher than that of RM, but lower than those of hybrid hens; during the laying period, ER laid eggs with a thinner eggshell [19], and a lower albumen HU in comparison to the other genotypes [18]. In a recent trial, the ER thickness showed low values also under a rearing environmental temperature of about 12 °C, both at 36 and 50 weeks of age [14]: this fact indicates possible peculiarities of the eggshell formation in ER and further study is needed for this breed.

The Table 4 resumes the effect of age on albumen quality of 7 d eggs per each genotype. After 1 weeks of storage the eggs of ER, RM and HW showed no significant differences for the albumen height throughout the ages; HB showed a linear decrease (*p* < 0.05).

The HU changed in ER and HB, according to a linear (*p* < 0.01) decrease. HW and RM did not change for HU. The 7 d aged eggs showed a marked decrease (*p* < 0.05) of HU only at 43 weeks in ER and HB, as a consequence of the decreased weight for the loss of water from the albumen. In general, at 7 days of storage the eggs show a stable quality as the main changes have occurred throughout the first days after oviposition [37].

Table 5 shows the effect of the age on the albumen quality after 14 days of storage per each genotype. As far as albumen height is concerned, it decreased for ER (*p* < 0.01) and HW (*p* < 0.01) at 35 weeks, following a significant (*p* < 0.01) linear trend, whereas RM and HB did not change.

The HU decreased according to the age in ER (at 35 weeks, *p* < 0.01), HW (at 35 weeks, *p* < 0.01) and HB (at 39 weeks, *p* < 0.01), whereas RM did not change. A significant linear decrease for ER (*p* < 0.01), HB (*p* < 0.01) and HW (*p* < 0.01) was found. After 2 weeks of storage the responses of the eggs varied according to the genotypes; ER and HW eggs showed a decrease of quality, earlier than HB, probably for their thinner eggshells and a more relevant albumen deterioration and water loss.

When the quality of albumen, especially during storage, is studied, some characteristics of the egg and its shell have to be considered, such as the shape index and the shell colour and thickness. The ER eggs were less spherical in comparison with the eggs of the other groups and showed a higher surface area to volume ratio [19]. As far as the shape range (65–85%) of avian eggs is concerned, the shape of eggs laid by *Gallus gallus domesticus* hens is in the middle [38]. The normal or characteristic shape of the egg is determined in the magnum, but the specific shape is fixed by the shell membranes, established as the egg moves through the isthmus [39]. Among the factors affecting the final egg shape of the birds, some morphological traits, such as the morphology of the pelvis, abdomen or oviduct, associated with flight ability should be considered. Morphological traits such as a lower body weight and dimensions of the ER hens may justify a lower egg shape in comparison to that of the RM birds, whereas for the hybrids, selected traits for an intense ovulation rate and high albumen deposition have to be considered; more knowledge is needed to elucidate the eggshell formation and shape, especially in ER eggs. As far as the eggshell colour is concerned, a previous work indicated a wide variation of shell colour in eggs laid by pheasants (*Phasianus colchicus* L.), as the shell colour of their eggs can vary from very light to dark brown, for the presence of brown protoporphyrin which is derived from blood haeme [10]. The amount of the pigments synthesized in the uterus [40] and deposited in the shell is proportional to the length of time that the egg stays in the eggshell gland and the brightness of the shell color is negatively correlated with its thickness [10]. Our results agree with those of Nowaczewski et al. [10] as, among the brown-eggshell genotype, ER eggs had a shell less pigmented (light brown) than those of HB and RM (dark brown). The eggshell thickness is involved in exchange of gases and water vapor conductance and thus it affects the hatchability of the fertilized eggs, but it is also important in table eggs as being involved in chemical reactions and water loss from the albumen during storage time. In fact, the oxidation reactions in the eggs, which take place during storage, are caused by entry of air through the pores in the shell, and this lead to an increase in albumen pH [41], loss of carbohydrates from the ovomucin molecule, breakdown in the gel structure of the thick layer [42] and water evaporation [43]. The loss of carbohydrate units that are linked o-glycosidically in ovomucin [44] leads to a breakdown of the ovomucin-lysozyme complex and causes a thinning of albumen and a decrease in Haugh Units [45].

In Table 6 the effect of age on the albumen quality after 21 days of storage per each genotype is shown. Throughout the period considered, the albumen height significantly changed according to the age in three groups: in ER and HW it decreased at 35 weeks (*p* < 0.01) and then gradually increased; the same trend was observed in HB (*p* < 0.05).

These three groups showed variations according to a significant (*p* < 0.01) quadratic trend. RM did not change. All the three genetic groups showed lower values at 35 weeks in comparison to 31 weeks and an increase until 43 weeks, but at this latter age ER did not show higher values than those at 35 weeks, as the hybrids did. This trend is different from that observed for 14 d eggs for ER and hybrid genotypes and suggests that other factors besides age and total egg mass can affect the quality of the albumen and its variation during storage. As stated above, the ER eggs are more ovoid than the eggs of the other genotypes and this trait, along with other physical characteristics, may affect the internal quality of an egg after laying. The egg shape should limit water loss and also permit gaseous exchange according to a good balance. The environmental conditions, where the laying activity of the birds occurs, affect the eggshell conductance which is relatively low in eggs of birds living in deserts, at high altitudes, or at high temperatures [46,47], but it has to allow an effective exchange of oxygen and carbon dioxide between the embryo and the atmosphere. For any given volume, an ovoid egg has a higher surface area to volume ratio than does a spherical egg. Therefore, all else being equal, an ovoid egg will gain and lose heat less slowly, lose more water, and have higher exposure to solar radiation than will a more ovoid egg with the same volume [48]. It is worth remembering that the lowest albumen height has been observed after a period with high environmental temperature, and the hens could have exhibited a particular physiological response involving also the albumen proteins expression and production [49]. The HU showed decreasing values from 35 weeks and according to the age for ER, HB, and HW, whereas RM unchanged. For ER a significant decreasing linear trend was observed (*p* < 0.01), whereas for the hybrids the changes followed a quadratic trend (HB: *p* < 0.01; HW: *p* < 0.01). After 35 weeks ER showed an increase of albumen height and a decrease of HU, respectively, less and more marked in comparison to those of the hybrid groups, because of the condition of its eggshell thickness which decreased at 39 weeks of age.

**Figure 4:**
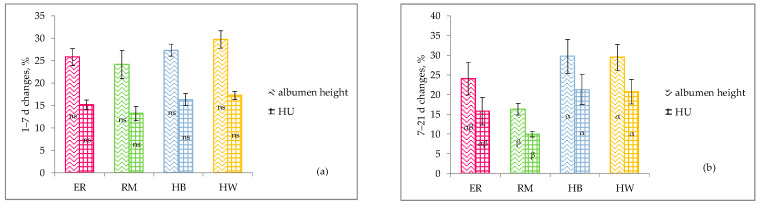
Effect of genotype ^1^ on changes (%, lsmeans ± SE) of albumen height ^2^ and Haugh Units ^3^ (HU) from 1 to 7 days (**a**) and from 7 to 21 days (**b**) of storage. Different letters among similar columns (trait, expressed as loss) indicate different values: ^α, β^: *p* < 0.10. ns: no significance. ^1^ Genotype: ER: Ermellinata di Rovigo; RM: Robusta maculata; HB: Hy-Line Brown; HW: Hy-Line White 36. ^2, 3^ Observations (*n*) for all the genotpes: 4 (at each age).

As far as the albumen quality from 1 day until 21 days of storage is concerned, in Figure 4 the effect of genotype on the percentage decreases for albumen height and HU during storage is shown. In the first 7 days of storage (Figure 4a), the four groups did not show different changes in albumen height and HU, but from the second until the third week of storage (Figure 4b) the changes were lower in the in RM (*p* < 0.10) than in the hybrids, and ER was intermediate.

The analysis of the decrease of the egg weight throughout the storage time along the studied period (Figure 2) showed that the purebred eggs lost weight, as water loss, almost at each age (Figure 2a, ER at 31, 35, 39 weeks: 7.2%, 5.2% and 6.2%, respectively, *p* < 0.01; Figure 2b, RM at 31 and 39 weeks: 5.1% and 4.4%, respectively, *p* < 0.05), whereas the hybrids had a significant (*p* < 0.01) decrease of egg weight only at 31 weeks (Figure 2c, HB: 5.7%; Figure 2d, HW: 5.3%). Thereafter, HB and HW eggs did not show any significant loss weight due to possible reactions involving also the passage of water across the vitelline membrane; at 43 weeks their weight loss (3.8%) tendentially overcame those of ER (2.4%) and RM (0%), and the water loss was lower than those of the previous ages. For this last result, the changed environmental temperature conditions throughout the trial, especially from the time of egg laying until the time of the egg collection from the nests, which are responsible for the initial chemical reactions inside the egg, and/or the changed chemical composition of the egg components inside the oviduct, should be considered also. The decrease of the egg weight, as a consequence of water loss from the eggshell, can be due to the eggshell traits, such as number and dimensions of pores, eggshell thickness, eggshell membranes and shape and surface area to volume ratio, as indicated above. ER showed a shape more ovoidal and a higher surface to volume ratio than those of the other groups [19], and the shape decreased with age [14] with a higher surface exposed to the external environment.

**Figure 5:**
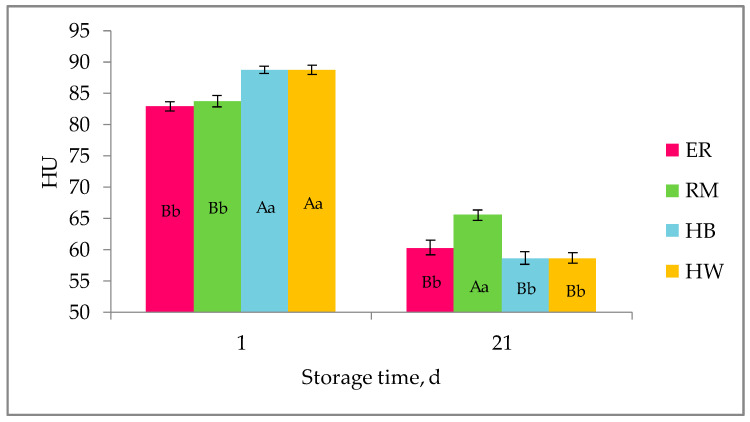
Effect of genotype ^1^ on Haugh Units (HU, lsmeans ± SE) according to storage time throughout the laying period. Different letters among columns per each storage time indicate different values: a, b: *p* < 0.05; A, B: *p* < 0.01. ^1^ Genotype: ER: Ermellinata di Rovigo; RM: Robusta maculata; HB: Hy-Line Brown; HW: Hy-Line White 36. ^2^ Observations (*n*) at 1 d: ER, HB, HW (114), RM (93); at 21 d: ER (92), RM (76), HB (104), HW (100).

In Figure 5, the effect of genotype on the HU of fresh and stored eggs, referred to the total laying period, is shown. The 1 d hybrid eggs showed an albumen quality (HU) higher (*p* < 0.01) than those of 1 d purebred eggs, and after 7 days of storage, differences between purebreds and hybrids disappeared (data not shown); at 21 d of storage, the quality did not differ among the groups, with the exception of RM, which was higher than the others. It seems that for an evaluation of albumen quality by HU, the comparison among the four genotypes needs a comment, as purebred and hybrid eggs differ for albumen proportion and yolk to albumen ratio, as a consequence of selection for higher albumen weight in hybrids, and separated comparisons between purebreds and between hybrids should be considered.

In Table 7 the correlation between the albumen pH and albumen height in fresh and stored eggs is shown. A significant negative correlation between albumen pH and height was detected in 1 d egg of all the genotypes, particularly in RM and hybrids (*p* < 0.01). In the stored eggs, a negative correlation was observed in HW, at each storage time, and in ER, after 14 and 21 days of storage. The HW and ER results indicate an entry of air through the pores in the shell during 21 days of storage and consequent oxidation reactions in the eggs and an increase in albumen pH [41], whereas for RM and HB, with a more coloured eggshell and higher eggshell thickness, these reactions seemed to be lower or other physiological and chemical responses seems to have occurred after one day of storage. Passages of water between yolk and albumen across the vitelline membrane, as stated by other authors [37] on hybrid eggs, could be responsible for the lack of correlations between albumen pH and height. Further studies are needed to elucidate how the egg components change according to the storage time and affect the egg quality according to the genotype and age.

## 4. Conclusions

Nowadays, egg production based on purebred hens is a very negligible part of the total egg mass of an industrialized country, but its importance is increasing both for biodiversity purposes and for a further opportunity to provide a choice of table eggs for the consumer. It is important to know the quality of eggs laid by hens reared outdoors under different environmental conditions throughout the productive cycle, as they can affect the internal changes of components in fresh and stored eggs. Quality indications on the egg cartons could be useful for the consumer who is interested in a higher quality during storage and for particular cooking preparations.

The results of this trial and those of previous works [14,19] add knowledge to the quality profile of fresh and stored eggs produced by purebred and hybrid hens, reared outdoors. In this trial, where genotypes, different for some phenotypical and physiological traits, were studied in the presence of variable environmental conditions, the effect of age on egg production and quality has to be considered as not a total intrinsic factor, as interactions between birds and environment may have occurred to a different extent according to genotype. The genotypes showed differences in egg production and fresh and stored egg quality throughout the laying cycle. RM showed quite a stable egg and albumen quality and a lower total egg mass than ER which showed a more variable albumen quality, due to a possible effect of lower eggshell thickness and shape index. The hybrids produced a higher total egg mass and a more stable curve than the purebreds and showed an intermediate variation of the egg quality, with an albumen quality, evaluated as HU, higher than that of ER and RM only in 1 d eggs as a result of a higher albumen weight. More studies are needed for understanding how the hen’s genotype modify their physiological responses in egg formation according to the phase of oviposition and to the environmental conditions, involving the albumen quality and affecting its changes during storage.

## Figures and Tables

**Table 1:** Environmental temperature and relative humidity, and productive performance of purebred and hybrid genotypes throughout the laying period.

Items	Environmental Conditions				Genotypes ^1^			
	Temperature, °C			RH, %	ER	RM	HB	HW
Laying Period	Average	Min	Max	Average	Cumulative egg mass, kg			
26–30 weeks	28	18	32	63	0.92	0.27	1.31	1.47
31–34 weeks	21	10	24	64	1.81	0.95	2.52	2.79
35–38 weeks	19	11	24	83	2.65	1.72	4.12	4.25
39–42 weeks	10	5	12	69	3.42	2.75	5.80	5.78
					Hen-day egg production, %			
25 weeks					47.8	4.4	81.2	78.9
25–44 weeks					56.3	45.6	85.2	85.8
					Egg mass/body gain, g/g			
25–44 weeks					9.6	4.4	17.6	34.3

^1^ Genotype: ER: Ermellinata di Rovigo; RM: Robusta maculata; HB: Hy-line brown; HW: Hy-line white. RH = relative humidity.

**Table 2:** Effect of age on eggshell thickness and weight, and albumen weight of 1 d eggs according to the genotype of the hens.

Items	Genotypes ^1^			
	ER	RM	HB	HW
Eggshell thickness ^2^, μm				
31 weeks	338 ^a^	372 ^Aa^	376	342 ^AaBb^
35 weeks	339 ^a^	342 ^Bb^	368	329 ^Bb^
39 weeks	315 ^b^	368 ^Aa^	366	332 ^ABb^
43 weeks	320 ^a^	383 ^Aa^	364	349 ^Aa^
*p*-value	0.0135	<0.0001	0.2395	0.0065
RMSE	33.14	25.67	23.02	23.85
component	linear *p* = 0.007	quadratic *p* = 0.0001	ns	quadratic *p* = 0.0009
Eggshell weight ^3^, g				
31 weeks	5.15 ^b^	5.67 ^b^	6.48 ^b^	5.72 ^b^
35 weeks	5.30 ^ab^	5.92 ^b^	6.79 ^a^	6.00 ^b^
39 weeks	5.65 ^a^	6.73 ^a^	6.77 ^a^	6.66 ^a^
43 weeks	5.60 ^a^	6.80 ^a^	6.91 ^a^	6.60 ^a^
*p*-value	0.0084	<0.0001	0.0226	<0.0001
RMSE	0.62	0.56	0.55	0.55
Component	linear *p* = 0.0016	linear *p* < 0.0001	linear *p* = 0.0056	linear *p* < 0.0001
Albumen weight ^4^, g				
31 weeks	31.9 ^Bb^	32.3 ^Bb^	37.1 ^Bb^	36.2 ^Bc^
35 weeks	32.7 ^AaBb^	33.7 ^AaBb^	41.4 ^Aa^	38.7 ^ABb^
39 weeks	32.0 ^Bb^	34.9 ^Aa^	41.5 ^Aa^	38.6 ^ABb^
43 weeks	34.6 ^Aa^	35.5 ^Aa^	42.5 ^Aa^	40.0 ^Aa^
*p*-value	0.0020	< 0.0001	< 0.0001	0.0001
RMSE	2.91	2.27	3.26	3.11
Component	linear *p* = 0.0030	linear *p* < 0.0001	linear *p* < 0.0001	linear *p* < 0.0001

Different letters among rows for each genotype indicate different values: a, b, c: *p* < 0.05; A, B: *p* < 0.01. RMSE: Root Mean Squared Error. ns: no significance. ^1^ Genotype: ER: Ermellinata di Rovigo; RM: Robusta maculata; HB: Hy-Line Brown; HW: Hy-Line White 36. ^2^ Observations (*n*) per age (at 31, 35, 39, 43 weeks): ER (29, 30, 26, 26), RM (20, 20, 28, 28); HB (30, 30, 29, 30); HW (30, 30, 29, 29). ^3^ Observations (*n*) per age (at 31, 35, 39, 43 weeks): ER (30-32-25-27), RM (25, 15, 30, 29); HB (30, 30, 30, 30); HW (30, 35, 30, 28). ^4^ Observations (*n*) per age (at 31, 35, 39, 43 weeks): ER (30, 30, 26, 28), RM (25, 20, 30, 29); HB (30, 30, 30, 30); HW (30, 29, 30, 30).

**Table 3:** Effect of age on albumen height and Haugh Units of 1 d egg according to the genotype of the hens.

Items	Genotypes ^1^			
	ER	RM	HB	HW
Albumen height ^2^, mm				
31 weeks	6.93 ^AaBb^	7.10	7.99 ^AaBb^	8.08 ^ab^
35 weeks	6.57 ^BbCc^	7.20	8.19 ^AaB^	8.36 ^a^
39 weeks	7.53 ^Aa^	7.27	8.63 ^Aa^	8.15 ^ab^
43 weeks	6.09 ^Cc^	6.47	7.45 ^Bb^	7.38 ^b^
*p*-value	<0.0001	0.0797	0.0006	0.0308
RMSE	1.02	1.16	1.02	1.34
Component	cubic *p* < 0.0001	ns	quadratic *p* = 0.0005	linear *p* = 0.0345
Haugh Units ^3^				
31 weeks	85.3 ^AaBb^	85.4	90.0 ^Aa^	90.7 ^Aa^
35 weeks	81.6 ^BbCc^	86.0	89.9 ^Aa^	91.1 ^Aa^
39 weeks	88.4 ^Aa^	84.2	91.2 ^Aa^	89.2 ^AaBb^
43 weeks	77.5 ^Cc^	79.8	84.1 ^Bb^	84.4 ^Bb^
*p*-value	<0.0001	0.0690	<0.0001	0.0025
RMSE	6.67	8.66	5.53	7.48
component	cubic *p* < 0.0001	ns	linear *p* = 0.0004	linear *p* = 0.0010

Different letters among rows for each genotype indicate different values. a, b, c: *p* < 0.05; A, B, C: *p* < 0.01. RMSE: Root Mean Squared Error. ns: no significance. ^1^ Genotype: ER: Ermellinata di Rovigo; RM: Robusta maculata; HB: Hy-Line Brown; HW: Hy-Line White 36. ^2, 3^ Observations (*n*) per age (at 31, 35, 39, 43 weeks): ER (30, 30, 24, 30), RM (25, 22, 22, 24); HB (30, 30, 24, 30); HW (30, 30, 24, 30).

**Table 4:** Effect of age on albumen height and Haugh Units of stored 7 d eggs according to the genotype of the hens.

Items	Genotypes ^1^			
	ER	RM	HB	HW
Albumen height ^2^, μm				
31 weeks	5.18	5.07	5.99 ^a^	5.52
35 weeks	5.05	5.44	6.10 ^a^	5.63
39 weeks	5.17	5.22	5.95 ^a^	5.69
43 weeks	4.65	5.49	5.39 ^b^	5.60
*p*-value	0.2456	0.3616	0.0493	0.9449
RMSE	1.02	0.86	0.97	1.00
component	ns	ns	linear *p* = 0.0258	ns
Haugh Units ^3^				
31 weeks	73.9 ^Aa^	73.1	78.2 ^Aa^	75.6
35 weeks	70.9 ^AaCc^	73.5	76.5 ^Aa^	73.2
39 weeks	73.2 ^AaCc^	71.2	74.4 ^AaB^	73.7
43 weeks	64.7 ^BbC^	73.0	68.5 ^Bb^	71.6
*p*-value	0.0045	0.7144	0.0002	0.3596
RMSE	9.44	6.84	7.84	7.83
component	linear *p* = 0.0040	ns	linear *p* < 0.0001	ns

Different letters among rows for each genotype indicate different values. a, b, c: *p* < 0.05; A, B, C: *p* < 0.01. RMSE: Root Mean Squared Error. ns: no significance. ^1^ Genotype: ER: Ermellinata di Rovigo; RM: Robusta maculata; HB: Hy-Line Brown; HW: Hy-Line White 36. ^2, 3^ Observations (*n*) (at 31, 35, 39, 43 weeks): ER (25, 25, 16, 25), RM (20, 18, 21, 25); HB (25, 25, 25, 25); HW (24, 26, 25, 25).

**Table 5:** Effect of age on albumen height and Haugh Units of stored 14 d eggs according to the genotype of the hens.

Items	Genotypes ^1^			
	ER	RM	HB	HW
Albumen height ^2^, μm				
31 weeks	4.97 ^Aa^	4.63	4.94	5.35 ^Aa^
35 weeks	4.12 ^Bb^	5.08	5.22	4.57 ^Bb^
39 weeks	4.05 ^Bb^	4.81	4.55	4.69 ^ABb^
43 weeks	3.82 ^Bb^	4.87	4.62	4.56 ^Bb^
*p*-value	0.0007	0.5395	0.0571	0.0025
RMSE	0.68	1.00	0.88	0.71
component	linear *p* = 0.0001	ns	ns	linear *p* = 0.0026
Haugh Units ^3^				
31 weeks	72.8 ^Aa^	69.5	70.6 ^Aa^	74.2 ^Aa^
35 weeks	63.2 ^Bb^	71.1	69.5 ^Aab^	65.5 ^Bb^
39 weeks	61.2 ^Bbc^	67.8	61.4 ^Bc^	65.0 ^Bb^
43 weeks	57.1 ^Bc^	69.1	62.7 ^ABbc^	61.5 ^Bb^
*p*-value	<0.0001	0.7579	0.0009	<0.0001
RMSE	6.64	8.24	8.52	6.54
component	linear *p* < 0.001	ns	linear *p* = 0.0005	linear *p* < 0.0001

Different letters among rows for each genotype indicate different values. a, b, c: *p* < 0.05; A, B, C: *p* < 0.01. RMSE: Root Mean Squared Error. ns: no significance. ^1^ Genotype: ER: Ermellinata di Rovigo; RM: Robusta maculata; HB: Hy-Line Brown; HW: Hy-Line White 36. ^2, 3^ Observations (*n*) per age (at 31, 35, 39, 43 weeks): ER (17, 20, 17, 18), RM (17, 15, 15, 16); HB (18, 20, 23, 20); HW (18, 20, 23, 20).

**Table 6:** Effect of age on albumen height and Haugh Units of stored 21 d eggs according to the genotype of the hens.

Items	Genotypes ^1^			
	ER	RM	HB	HW
Albumen height ^2^, μm				
31 weeks	4.38 ^Aa^	4.26	4.25 ^ab^	4.24 ^Aa^
35 weeks	3.34 ^Bb^	4.44	3.79 ^b^	3.51 ^Bb^
39 weeks	3.74 ^AaBb^	4.25	3.92 ^ab^	3.87 ^AaBb^
43 weeks	3.75 ^AaBb^	4.82	4.43 ^a^	4.19 ^Aa^
*p*-value	0.0003	0.1013	0.0148	0.0006
RMSE	0.85	0.81	0.78	0.67
component	quadratic *p* = 0.0052	ns	quadratic *p* = 0.0019	quadratic *p* = 0.0002
Haugh Units ^3^				
31 weeks	68.0 ^Aa^	66.1	64.1 ^Aa^	64.8 ^Aa^
35 weeks	54.1 ^Bb^	65.9	54.5 ^Bb^	52.7 ^Cc^
39 weeks	59.2 ^ABb^	63.0	54.7 ^Bb^	56.3 ^BbCc^
43 weeks	56.6 ^Bb^	67.1	60.3 ^AaBb^	59.4 ^AaBb^
*p*-value	<0.0001	0.3439	0.0003	<0.0001
RMSE	9.86	7.46	9.33	7.40
component	linear *p* = 0.0018	ns	quadratic *p* < 0.0001	quadratic *p* < 0.0001

Different letters among rows for each genotype indicate different values. a, b, c: *p* < 0.05; A, B, C: *p* < 0.01. RMSE: Root Mean squared error. Ns: no significance. ^1^ Genotype: ER: Ermellinata di Rovigo; RM: Robusta maculata; HB: Hy-Line Brown; HW: Hy-Line White 36. ^2, 3^ Observations (*n*) per age (at 31, 35, 39, 43 weeks): ER (30, 24, 18, 20), RM (20, 16, 20, 20); HB (29, 25, 25, 25); HW (28, 24, 24, 24).

**Table 7:** Pearson’s correlation between albumen pH and height in fresh and stored eggs.

Items	Genotypes ^1^							
	ER		RM		HB		HW	
	r	*p*-Value	r	*p*-Value	r	*p*-Value	r	*p*-Value
1 d egg	−0.19	0.04	−0.38	0.0008	−0.29	0.002	−0.41	<0.0001
7 d egg	0.03	0.75	−0.14	0.22	−0.05	0.59	−0.27	0.006
14 d egg	−0.51	<0.0001	−0.08	0.54	−0.14	0.22	−0.46	<0.0001
21 d egg	−0.45	<0.0001	0.06	0.64	−0.12	0.23	−0.28	0.005

^1^ Genotype: ER: Ermellinata di Rovigo; RM: Robusta maculata; HB: Hy-Line Brown; HW: Hy-Line White 36. Observations (*n*) per storage time (at 1 d, 7 d, 14 d, 21 d): ER (112, 85, 66, 88), RM (86, 77, 60, 70); HB (112, 100, 78, 100); HW (112, 100, 77, 98).

## Data Availability

Data presented are original and not inappropriately selected, manipulated.
